# Elastin content is high in the canine cruciate ligament and is associated with degeneration

**DOI:** 10.1016/j.tvjl.2013.11.002

**Published:** 2014-01

**Authors:** K.D. Smith, P.D. Clegg, J.F. Innes, E.J. Comerford

**Affiliations:** a Faculty of Veterinary Medicine, Small Animal Hospital, University of Glasgow, 464 Bearsden Road, Glasgow G61 1QH, UK; b Department of Musculoskeletal Biology, Institute of Ageing and Chronic Disease, Faculty of Health and Life Sciences, University of Liverpool, Liverpool CH64 7TE, UK

**Keywords:** Canine, Cruciate ligament, Elastin, Degeneration, Oxytalan

## Abstract

Cruciate ligaments (CLs) are primary stabilisers of the knee joint and canine cranial cruciate ligament disease (CCLD) and rupture is a common injury. Elastin fibres, composed of an elastin core and fibrillin containing microfibrils, are traditionally considered minor components of the ligament extracellular matrix (ECM). However, their content and distribution in CLs is unknown. The purposes of this study were to determine the elastin content of canine CLs and to ascertain its relationship to other biochemical components and histological architecture.

Macroscopically normal CLs were harvested from Greyhounds (*n* = 11), a breed with a low risk of CCLD. Elastin, collagen and sulfated glycosaminoglycan content were measured and histological scoring systems were developed to quantify ECM changes using a modified Vasseur score (mVS) and oxytalan fibre (bundles of microfibrils) staining. Elastin contents were 9.86 ± 3.97% dry weight in the cranial CL and 10.79 ± 4.37% in the caudal CL, respectively, and did not alter with advancing histological degeneration. All CLs demonstrated mild degenerative changes, with an average mVS score of 11.9 ± 3.3 (maximum 24). Increasing degeneration of the ligament ECM showed a positive correlation (*r* = 0.690, *P* < 0.001) with increased oxytalan fibre staining within the ECM.

Elastin is an abundant protein in CLs forming a greater proportion of the ligament ECM than previously reported. The appearance of oxytalan fibres in degenerative CL ECM may reflect an adaptive or reparative response to normal or increased loads. This finding is important for future therapeutic or ligament replacement strategies associated with cranial CL injury.

## Introduction

Cruciate ligaments (CLs) are the primary stabilisers of the knee joint. The CL complex is comprised of the cranial cruciate ligament (CCL) wrapping around the caudal cruciate ligament (CaCL) (Arnoczky and Marshall, 1977). Canine CCL disease (CCLD) is a progressive degenerative process (Bennett et al., 1988) and ligament rupture typically occurs through a non-contact injury (Prodromos et al., 2007, Renstrom et al., 2008). The CCL will not heal following rupture and attempts at primary repair have been unsatisfactory (Feagin and Curl, 1976).

Non-contact CCL injury has been reported in humans (Serpell et al., 2011) as well as in dogs (Comerford et al., 2005), and alterations to the CCL extracellular matrix (ECM) and cellular metabolism are implicated in its pathogenesis. A recent study demonstrated degenerative histological changes in the human anterior cruciate ligament (ACL) without cartilage lesions (Hasegawa et al., 2012). These findings suggested that ACL degeneration may precede cartilage damage in certain individuals and therefore play an important role in the pathogenesis of osteoarthritis (OA).

Pedigree dog breeds provide a valuable opportunity to access CL tissue alongside well-documented divergent breed predispositions to CCL injury (Whitehair et al., 1993). They therefore represent ideal species in which to examine ECM changes in mammalian CCLs. In dogs, increased ECM degeneration and collagen turnover have been observed in the CLs of breeds at a high risk of CCL failure (e.g. Labrador retriever) when compared to low risk breeds such as the Greyhound (Comerford et al., 2005, Comerford et al., 2006b).

Although collagen provides tensile strength to the ligament, other structural components, such as elastin, most likely contribute to the overall mechanical function of the complex (Frank, 2004, Ujiie et al., 2008). A recent study suggested that elastin may play an important role in the toe-region of the stress strain curve by helping to pre-stress and stabilise collagen fibrils in ligaments (Henninger et al., 2013). Elastin has been considered a minor component of ligament ECM (Frank, 2004) but has never been quantified in the CL complex in any species, and its role in ligament ECM is unknown. Elastin was shown to comprise 13.8% of the caudal dorsal ligament (Nakagawa et al., 1994) and up to 9.3% of the annulus fibrosus in man (Cloyd and Elliott, 2007).

Elastin fibres (EFs) are comprised of an elastin core within a fibrillin-containing microfibril (MF) scaffold imparting extensibility and resilience to soft tissues (Kielty, 2006). Bundles of MFs are known as oxytalan fibres (OFs) and, collectively, MFs, OFs and EFs are known as elastic fibres. Failure of elastic fibres has been implicated in a number of serious conditions, such as congenital contractile arachnodactyly and Marfan syndrome (Kielty, 2006), and changes in elastic fibres have been associated with degeneration in the annulus fibrosus (Cloyd and Elliott, 2007). Although the role of EFs in the CL complex is not well understood, recent studies have proposed a role in collagen reorganisation following ligament deformation (Smith et al., 2011).

In this study, our objective was to determine the content and distribution of elastin in healthy canine CLs, and its relationship with any degenerative ligament changes. To fulfil this objective we asked the following questions: (1) What is the elastin content in CLs in comparison to other major ECM proteins (collagen and sulfated glycosaminoglycans [sGAG])? (2) What is the elastic fibre distribution within the CL architecture and does it alter with degenerative changes, if present? (3) Is there a relationship between biochemical (elastin content) and histological (elastic fibre distribution and CL architecture) parameters? (4) Are there any differences between CCLs and CaCLs?

## Materials and methods

This study was designed as an experimental descriptive study determining elastin in healthy CLs. Eleven pairs of CLs (CCL and CaCL) (*n* = 6 males and *n* = 5 females) were harvested from seven skeletally mature ex-racing Greyhounds (32–60 months old; mean, 42 ± 9.7; median, 39) with no macroscopic evidence of knee pathology. Eight dogs had recently been in racetrack training, two had not trained for 3 months and one dog had not trained for 6 months. The animals were euthanized for reasons other than musculoskeletal disease and informed owner consent was obtained in each case prior to tissue removal. Institutional ethical approval was not required at the time of data collection because owner consent was considered sufficient to use the cadaveric clinical waste material for research.

Each CL was sectioned into proximal, middle and distal sections of equal length. Of each of these subdivisions, further division through longitudinal sectioning into thirds of equal width allowed one-third to be formalin fixed for histology and the remaining two-thirds were stored at −80 °C until required for biochemical analysis. Elastin, collagen and sGAG content in the CLs were measured using biochemical assays. Samples were weighed, freeze-dried overnight at −60 °C and reweighed again to obtain percentage dry weight.

Elastin was measured using the Fastin dye-binding assay (Biocolor). Insoluble cross-linked elastin was converted to a soluble form (α- and κ-elastin polypeptides) by heating the ligament to 95 °C in 0.25 M oxalic acid (35295, Sigma–Aldrich) for 1 h. They were centrifuged at 3000 *g* for 10 min and the supernatant extracted. Preliminary analysis showed this needed to be repeated six times to extract all elastin. Pooled extracts were incubated with the dye 5,10,15,20-tetraphenyl-21,23-porphine tetrasulfonate for 90 min to bind α- and κ-elastin, lathyrogenic elastins, and soluble tropoelastin. Following centrifugation (10,000 *g*) for 10 min, the residue was resuspended in a dissociation agent (guanidine HCl and propan-1-ol) and absorbance read in a micro-well plate reader (Multiskan EX, Therma) at 440 nm. Samples were analysed in quadruplicate for each sample analysed. The standard used was a high molecular weight fraction of α-elastin (1.0 mg/mL in 0.25 M oxalic acid) prepared from bovine neck ligament elastin and provided with the assay kit. A standard curve was prepared for each assay.

Ligament samples were digested for 24 h with papain (300 μg/mL, P4762, Sigma–Aldrich) in phosphate-buffered saline (PBS) with 5 mM Cysteine HCl and 5 mM EDTA at 60 °C in preparation for measuring collagen and sGAG content. Collagen was measured using a colorimetric assay to determine hydroxyproline (OHPro) content (Jamall et al., 1981) assuming 13.7% of total collagen as OHPro. Briefly, samples were hydrolysed in 6 N HCl for 24 h at 110 °C before freeze-drying. Following reconstitution in water, quadruplicate aliquots were thoroughly mixed with a solution containing sodium acetate trihydrate, tri sodium citrate dehydrate, citric acid, propan-2-ol and chloramine T. The colour reagent, containing dimethylamino benzaldehyde, perchloric acid and propan-2-ol was added, then the mixture was heated at 70 °C for 20 min. Standards from stock OHPro (trans-4-hydroxyproline, Sigma) were used to calculate the standard curve from 0 to 10 μg/mL. Total sGAG was assessed using the 1,9-dimethylmethylene blue (DMMB) dye binding assay. Quadruplicate aliquots of papain-digested ligament samples were immediately analysed at 540 nm following the addition of DMMB. The assay was calibrated by use of standards up to 40 μg/mL shark chondroitin sulfate (Sigma–Aldrich) and sGAG concentration was obtained by comparison with the standard curve (Farndale et al., 1986).

CL architecture and elastic fibre distribution were determined using histological methods. Sequential sections (4 μm) from paraffin-embedded samples were stained with haematoxylin and eosin (H&E) and Miller’s stain (which will stain both EFs and fine OFs). This series (three slides for each proximal, middle and distal section) allowed for assessment of tissue architecture, and identification of elastin microfibrils. Images were recorded on a dedicated microscope (Nikon Eclipse 80i). All sections were read by two observers blinded to section location or animal on two separate occasions at least 1 week apart.

H&E sections were assessed for signs of CL degeneration and the entire section was examined. All sections were graded 0–3 according to criteria previously described (Vasseur et al., 1985). A novel scoring system was developed to allow subdivision of the broad grade 1 category. Eight categories were identified. A score from 0 to 4 was given based on the extent of the changes (absent, isolated, <25%, <50%, >50%) for each factor giving a range of possible scores from 0 to 24. These results are referred to as modified Vasseur Score (mVS). Preliminary analysis had shown that a single slide from each section could be considered representative of changes throughout the section (data not shown).

A novel scoring system was developed to quantify changes in microfibril staining. Increased staining in interfascicular and interbundle regions, ligament substance (intrabundle), as well as the extent and degree of pericellular staining, could be awarded up to two points giving a score range of 0–10. These results are referred to as Miller’s Score (MS). Preliminary analysis had shown that a single slide from each section could be considered representative of changes throughout the section (data not shown).

Elastin, collagen and sGAG were normalised to total ligament dry weight. Additionally, elastin was normalised to collagen. All results are reported as means and standard deviations (SD) and percentage total ligament dry weight. A two-factor ANOVA was used where the two factors were CL (two levels: CCL and CaCL) and location (three levels: proximal, middle and distal) to assess differences in biochemical and histological data. Where significance at 5% was met, a Bonferroni post hoc test was applied and *t* tests were used for direct comparisons. Pearson’s correlations (*r*) were used to assess relationships between factors with significance set at 5%. Results are presented as mean values ± SD. Kendall’s coefficient of concordance was calculated for intra- and inter-observer concordance of both mVS and MS. Kendall’s coefficient of concordance ranges from 0 to 1 with values closer to 1 suggesting a high degree of concordance. Data were analysed using Minitab Statistical Software.

## Results

Elastin content was 9.86 ± 3.97% in the CCL and 10.79 ± 4.37% in the CaCL. There were no statistically significant differences in elastin content with regard to location (proximal, middle or distal), age, sGAG or percentage dry matter. Collagen content was 75.63 ± 3.89% in the CCL and 75.04 ± 4.17% in the CaCL and increased with age (*r* = 0.813, *P* < 0.001), but not with location or other biochemical parameters. sGAG content was 0.08 ± 0.02% in the CCL and 0.04 ± 0.01% in the CaCL. When the elastin, collagen and sGAG contents were combined, total ligament dry weight was 84.7 ± 4.68% (range, 72.4–94.7%).

**Fig. 1:**
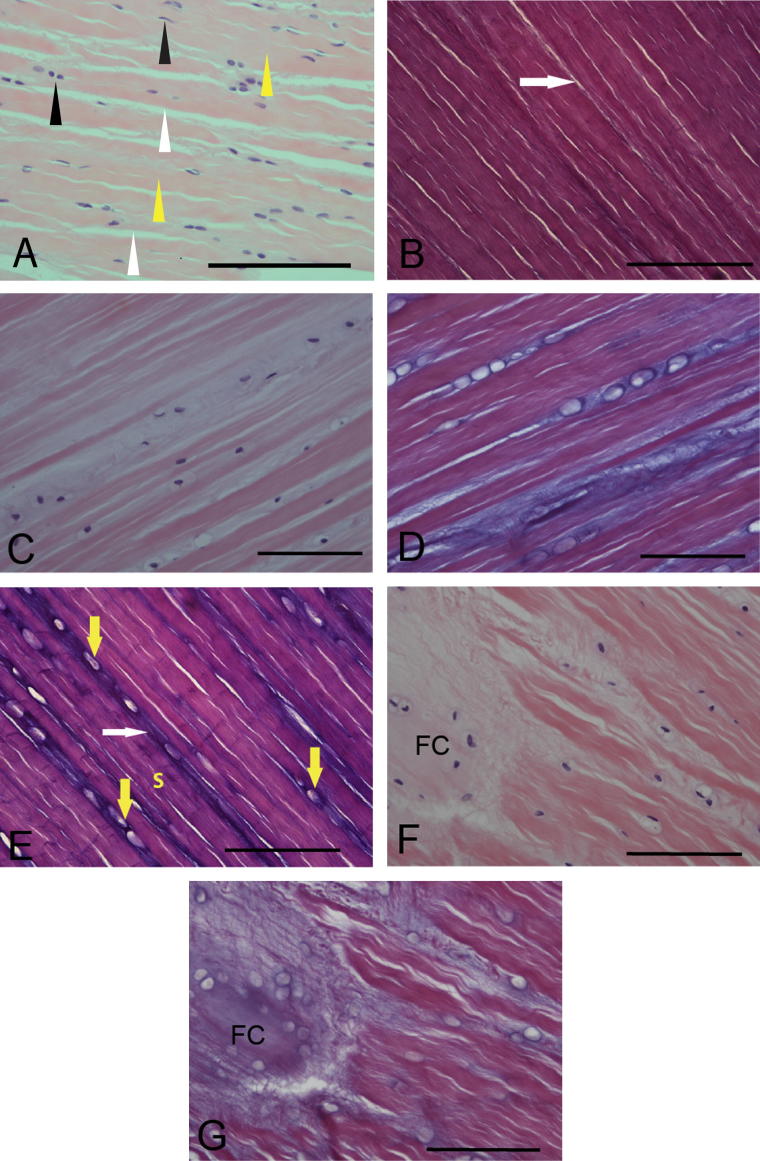
Histological changes in cruciate ligament (CLs) with mild degeneration. This image is from a section of normal Greyhound cranial cruciate ligament (CCL). There is a mixed cell population (spindle and oval, black arrows), regular staining of the collagen bundles (yellow arrows) and well defined interbundle areas (white arrows) (A) (Stain: H&E, CCL, ×40). Minimal oxytalan fibre (OF) staining (white arrow) is seen in this image of a section of normal Greyhound cranial cruciate ligament (CCL). (B) (Stain: Miller’s stain, CCL, ×40). Loss of cell density, collagen density and architecture along with degenerative change (pericellular halos) are demonstrated in this section (C) (Stain: H&E, CCL, x40). An image from same section as C shows marked pericellular oxytalan fibre staining of most degenerative cells, with moderate interbundle and substance staining (D) (Stain: Miller’s stain, CCL, ×40). Marked interbundle oxytalan fibre staining (white arrows) is seen along with widespread and marked pericellular staining (yellow arrows) and a moderate degree of CL substance stainings (E) (Stain: Miller’s stain, CCL, ×40). An area of complete loss of collagen architecture with mineralisation is seen and is considered to be fibrocartilaginous change (FC) (F) (Stain: H&E, CaCL, ×40). In this image from the same section as F, the area of fibrocartilaginous change (FC) shows a dense and fine meshwork of microfibrils (MFs) (G) (Stain: Miller’s stain, CaCL, ×40). Magnification bars, 100 μm.

All CL samples stained with H&E were graded as grade 1 according to the published system (Vasseur et al., 1985) and with an average score of 11.9 ± 3.3 (maximum score – 24) with the mVS system. In the majority of ligament there were no differences in either size or distribution of collagen or EFs (Fig. 1A and B), but in samples with reduced collagen architecture (Fig. 1C) there was a marked increase in OFs (Fig. 1D). Increased OF staining was differentially noted within three subdivisions of the ligament substance in all CLs with high grade 1 changes. Staining of OFs was loose and mesh-like in the interfascicular area. Cells that had a rounded appearance had increased pericellular OF staining (Fig. 1D and E). Interbundle staining of OFs showed marked differences between low and high mVS grade sections (Fig. 1D and E).

**Fig. 2:**
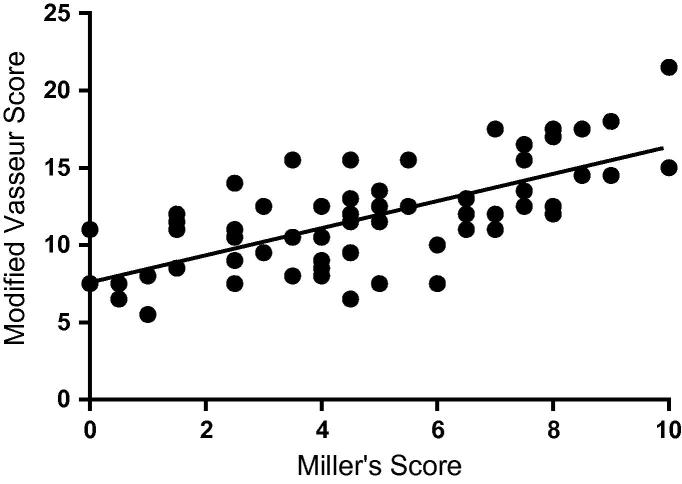
Relationship between modified Vasseur score (mVS) for H&E staining and Miller’s score (MS) for elastin microfibrils. A positive correlation between mVS and MS (*r* = 0.690, *P* < 0.001) in canine cruciate ligaments is demonstrated. As degeneration of the cruciate ligament advances, there is a tendency for increased microfibrillar staining.

Where collagen bundles remained largely intact, the OFs appeared as small fibres running parallel or obliquely to the collagen. If there was loss of collagen architecture, then these areas showed increased OF staining with less regular organisation (Fig. 1F and G). The average MS was 4.9 ± 2.0 for both CLs and was positively correlated with the mVS (*r* = 0.690, *P* < 0.001) (Fig. 2). Kendall’s coefficient of concordance for inter-observer concordance was 0.85 for mVS and 0.85 for MS. Intra-observer concordance for observer 1 was 0.94 (mVS) and 0.91 (MS) and for observer 2 was 0.95 (mVS) and 0.94 (MS).

**Fig. 3:**
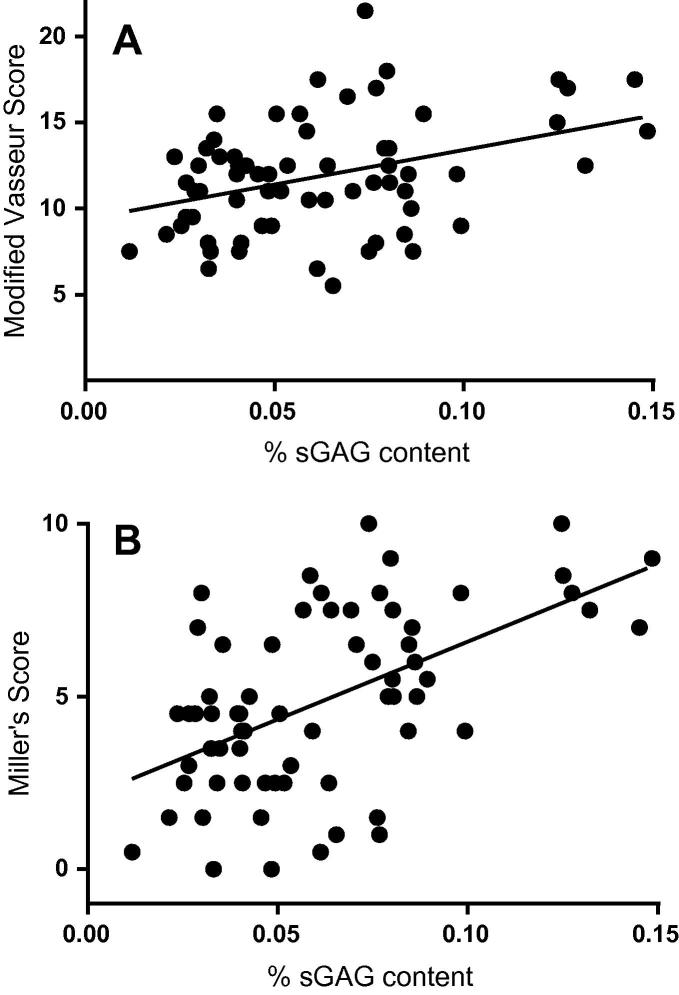
Relationship between biochemical and histological data in low risk CLs. (A) Sulfated glycosaminosglycans (sGAG) content correlates positively with the modified Vasseur score (*r* = 0.389, *P* < 0.002) indicating that as degeneration advances sGAG content increases. (B) sGAG content correlates positively with Miller’s score (*r* = 0.607, *P* < 0.001) indicating that as the score for elastin microfibrils increases so does sGAG content.

No correlations were noted between elastin and mVS (*r* = −0.016, *P* = 0.897) and MS (*r* = −0.105, *P* = 0.403) in the whole or ligament subsections, or between collagen and mVS (*r* = −0.061, *P* = 0.63) and MS (*r* = 0.04, *P* = 0.56). There was a positive correlation of sGAGs with mVS (*r* = 0.389, *P* = 0.002) (Fig. 3A) and MS (*r* = 0.607, *P* < 0.001) (Fig. 3B).

**Fig. 4:**
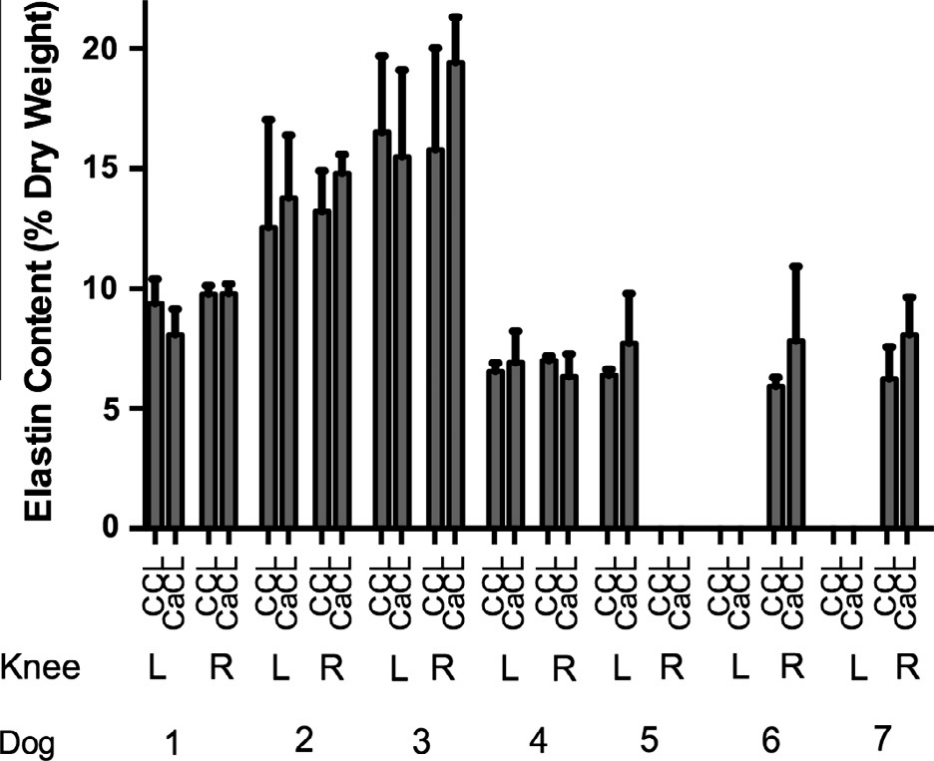
Elastin content of canine cruciate ligaments. Elastin averaged 9.86 ± 3.97% (total ligament dry weight) in the cranial cruciate ligament (CCL) and 10.79 ± 4.37% in the caudal cruciate ligament (CaCL) (*P* = 0.28). In dogs 1–4, both the left and right knees were studied and the pairs of CCLs and CaCLs had very similar intra- and inter-ligament elastin content. There were no significant statistical differences between the pairs of CCLs and CaCLs or between the left and right knees.

Although there were no statistically significant differences in elastin content between CCL and CaCL (*P* = 0.28), a marked variation in both ligaments was noted (range, 5.9–19.4%) (Fig. 4). Pairs of CCLs and CaCLs (from the same joint) had very similar intra- and inter-ligament elastin content, while sGAG contents were higher in the CCL (0.081 ± 0.019%) than the CaCL (0.043 ± 0.010%, *P* < 0.001). There were no significant differences in mVS, either within or between CCL and CaCL. A higher MS was seen in the CCL (5.3 ± 1.9) compared to the CaCL (4.0 ± 1.4, *P* = 0.02).

## Discussion

CCLD or non-contact injuries of the ACL are common in dogs (Bennett et al., 1988) and humans, respectively (Arendt and Dick, 1995). CCL injuries have major implications on affected animals’ quality of life as well as placing financial burdens on owners and pet insurance providers (Wilke et al., 2005). Many mechanisms of CCLD have been identified (Cook, 2010, Comerford et al., 2011). However, little is known about the role of ligament ECM proteins such as elastin in this condition. Determining the content and distribution of ECM proteins in the CCL is vital to understanding their role in CCLD.

Earlier studies have identified differences in collagen and sGAG content in canine CCLs (Comerford et al., 2006a). Other minor ECM components such as elastin may have a role in CL physiology and metabolism (Smith et al., 2011). It has been suggested that elastin may have a mechanical role in the CL complex with EFs absorbing recurrent maximal stresses, and OFs distributing complex multiaxial stresses through the collagen architecture (Strocchi et al., 1992).

Recent work has reported that elastin may be important in the toe region of the stress–strain curve of the porcine medial collateral ligament contributing to its viscoelastic properties (Henninger et al., 2013). This may provide an explanation for the high elastin content found in the CLs from exercising dogs in our study. Smith et al. (2011) have suggested a role for EFs in bundle reorganisation following CL deformation, a mechanism also proposed in the aortic valve (Vesely, 1998) and annulus fibrosus (Cloyd and Elliott, 2007, Yu et al., 2007). To date, the content and distribution of elastin in the CCL of any mammalian species has not been reported.

One limitation to our study was that the elastin assay used (i.e. Fastin) was unable to distinguish between insoluble elastin and degraded elastin peptides. It is likely that the elastin measured comprised both elastin and elastin peptides and monomers trapped within the ECM. Further work is required to determine whether the elastin measured is the tropoelastin precursor, insoluble elastin or degraded peptides. The negative correlation between elastin and collagen quantity suggests that the measured elastin results reflected ligament composition. The Fastin assay has been used in a number of tissues and species, where the results have been correlated with other biochemical methods of quantification (Romanowicz and Sobolewski, 2000, Cloyd and Elliott, 2007). Another limitation is the restricted age group of the dogs we examined and future work should aim to confirm our findings in dogs from a broader age range to better evaluate the relationship of age with biochemical parameters. Current work in our laboratory is seeking to confirm these findings with regard to elastin microfibrils using real time RT-PCR and immunohistochemistry.

We have shown that elastin is a significant ECM protein within the mammalian CCL, ranging from 5.9% to 19.4% ligament dry weight. Previous studies have estimated elastin content in the CL complex from 0% to 6% using histochemical staining of EFs, crosslink analysis (demosine and isodesmosine) or electron microscopy (Vasseur et al., 1985, Strocchi et al., 1992). These methods may have underestimated elastin content by only analysing intact EFs and not elastin precursors and degraded peptides. As EFs were considered sparse in the histological sections of the CLs examined in this study, this would suggest that not all elastin in CL tissue was contained within EFs and that other forms of elastin (precursors and/or degraded peptides) may have been measured by the Fastin assay.

Ultrastructural degeneration, including increased chondroid metaplasia, of non-diseased CCL ECM, has been reported in dog breeds at a high risk of CCL rupture (Comerford et al., 2006b). In the present study, OF staining appeared to increase with CL degeneration. Using novel histological scoring systems, we showed the increase in OFs to be proportional to the degree of degeneration. To date increased appearance of OFs with advancing degeneration has not been described in any ligament. OFs may have a number of roles in the CL complex including provision or maintenance of elasticity, stabilisation of blood vessels, anchoring tissue or guidance of cell migration (Chantawiboonchai et al., 1998, Tashiro et al., 2002). Assembly of OFs is commonly seen in healing responses in skin, artery (Sinha et al., 2001), muscle and myocardium (Vracko et al., 1990). OFs have been shown to contain both fibrillin 1 and fibrillin 2 in the canine CL (Smith et al., 2011) and further studies into the roles of these proteins in ligament pathophysiology are warranted.

In our study, we obtained ‘undiseased’, low-risk CLs from ex-racing Greyhounds but changes consistent with mild degeneration were found in every section examined. This may suggest that the histological changes observed were a physiological response to normal CL loading and/or exercise in this breed. Our findings indicate that the increased quantity of OFs observed in the CLs could reflect an adaptive or a reparative response to repeated microinjury as experienced with normal loading and/or exercise. Future work will involve the study of aged CLs as well as those with more advanced degeneration from dogs at high risk of CL rupture (Vasseur et al., 1985, Narama et al., 1996) to assess whether the increased OF staining is a feature of age and/or more advanced degeneration.

Increased collagen content with age was noted. Collagen fibril size distribution changes with age in all extraocular tissue contributing to increased strength (Parry et al., 1978). Furthermore, the mechanical environment can affect ligament structure and strength (Parry et al., 1978, Davankar et al., 1996) and exercise can induce local and systemic change to tendon and ligament (Woo et al., 1980, Woo et al., 1981, Viidik et al., 1996, Nielsen et al., 1998). Given that all of our dogs had been in training within the previous 6 months, we believe that the increase in collagen reflects both age and the influence of training.

The lack of correlation between elastin content and MS for OFs in the CCLs in this study suggest that the OF production is not associated with either elastin production, or subsequent development to EFs and that this process may be entirely fibrillin driven. A similar pattern has been described in the annulus fibrosus where degeneration was associated with a fivefold increase in elastin over that seen in healthy annulus fibrosus (Cloyd and Elliott, 2007) without increase in EFs (Olczyk, 1994, Smith and Fazzalari, 2006).

There were no significant differences between CCLs and CaCLs in terms of elastin content; however, CCLs had greater OF staining (MS) and sGAG contents. The increased MS in the midsubstance of the CCL was not mirrored in differences in mVS between CCLs and CaCLs, suggesting that increased OF staining may also be triggered by factors (such as a normal reparative response) other than those that lead to or precede degeneration. An increase in sGAG contents has been shown previously in ruptured CCLs compared to intact CCLs and was suggested to reflect change in ligament load (Comerford et al., 2004). Further work is required to understand whether these differences reflect intrinsic physiological differences or reflect differences in load and function.

## Conclusions

Elastin is a significant protein in CCLs from an exercising dog breed at a low risk of CCLD and rupture. The presence of OFs in these ligaments suggests that they may have an adaptive or reparative role secondary to normal or increased loads. These findings may be important in further elucidating the influence of altered loads on ligament ECM physiology, adaptation and repair in non-contact CCL injury in humans and comparative species.

## Conflicts of interest statement

None of the authors of this paper has a financial or personal relationship with other people or organisations that could inappropriately influence or bias the content of the paper.
